# SphK1 functions downstream of IGF-1 to modulate IGF-1-induced EMT, migration and paclitaxel resistance of A549 cells: A preliminary in vitro study

**DOI:** 10.7150/jca.32646

**Published:** 2019-07-10

**Authors:** Xingping Wu, Qibiao Wu, Xiqiao Zhou, Jianan Huang

**Affiliations:** 1 Department of Respiratory Medicine, the First Affiliated Hospital of Soochow University, Jiangsu, P.R. China; 2 State Key Laboratory of Quality Research in Chinese Medicines, Macau University of Science and Technology, Macau, P.R. China; 3 Department of Respirology, the First People's Hospital of Lianyungang, Jiangsu, P.R. China; 4 Department of Gastroenterology, the First Affiliated Hospital of Nanjing Medical University, Jiangsu, P.R. China

**Keywords:** SphK1, IGF-1, EMT, NSCLC, signaling pathway, therapeutic target.

## Abstract

Insulin-like growth factor-1 (IGF-1) -induced epithelial-mesenchymal transition (EMT) plays a key role in the metastasis and drug resistance of non-small cell lung cancer (NSCLC). Sphingosine kinase-1 (SphK1) is also involved in EMT of NSCLC. However, the interaction between SphK1 and IGF-1 in the EMT of NSCLC is largely unknown. To clarify this issue, we examined the involvement of SphK1 in IGF-1-induced EMT using human lung cancer cell line A549, and its paclitaxel-resistant subline. Cell viability was evaluated by cell counting kit-8 assay; Migratory ability was examined using scratch wound healing test; Protein expression levels of SphK1, vimentin, fibronectin, N-cadherin and E-cadherin were detected by western blot analysis, respectively. The results showed that, IGF-1 treatment of A549 cells stimulated the expression of SphK1, the activation of ERK and AKT, the cell migration, and the expression of EMT hallmark proteins, while inhibition of SphK1 by its specific inhibitor SKI-II suppressed all the above changes and increased the sensitivity of A549 cells to paclitaxel. Our data demonstrate that SphK1 acts as a downstream effector of IGF-1 and plays a critical role in IGF-1-induced EMT, cell migration and paclitaxel resistance of A549 cells, suggesting that SphK1 might be a potential therapeutic target for NSCLC.

## 1. Introduction

Worldwide, lung cancer remains the leading cause of cancer-related mortality, and approximately 85% of lung cancers are Non-small cell lung cancer (NSCLC) 1,2. The major causes of mortality in NSCLC patients are metastasis and drug resistance, which are closely associated with Epithelial-mesenchymal transition (EMT) 3-6. EMT is a process during which the epithelial cells lose their phenotype and acquire the characteristics of mesenchymal cells 5. Accumulating evidence shows that insulin-like growth factor (IGF) -1 signaling pathway is involved in EMT and IGF-1-induced EMT plays an important role in the development and progression of many types of solid tumors, including NSCLC 7-11.

Recently, sphingosine kinase (SphK) -1, an oncogenic kinase, has attracted increasing attention because of its important functions in many processes of cancer cells 12-14. A few studies showed that SphK1 might mediate the EMT of A549 cells, but the mechanisms remain ambiguous 15-16. Both IGF-1 and SphK1 have been shown to be involved in the EMT process of NSCLC. And a previous study provided the first illustration of SphK1 role as an crucial regulator of neuroblastoma cells' death by mediating IGF-1 17. These findings subsequently lead to a hypothesis that, there is probably a similar functional interaction between SphK1 and IGF-1 in the EMT process of NSCLC. However, little is known about this issue. In this study, we explored the role of SphK1 in IGF-1-induced EMT, migration and paclitaxel resistance of the human lung cancer cell line A549.

## Material and Methods

### 2.1 Cells and antibodies

A549 cell line was purchased from the American Type Culture Collection (Manassas, VA) and cultured in F-12K medium (Gibco) supplemented with 10% fetal bovine serum (FBS, Gibico), 100U/ml penicillin, and 100μg/ml streptomycin. Paclitaxel resistant-A549 was generated by culturing A549 cells in complete F-12K medium containing 2 μM paclitaxel. When indicated, cells were treated with IGF-1 (Peprotech) or SphK1 inhibitor SKI-II (Sigma Cat# S5696).

Antibodies used for immunoblot included anti-SphK1 (Cell Signaling Technology Cat# 3297), anti-phospho SphK1(Ser225) (ECM Biosciences Cat# SP1641), anti-Fibronection (Abcam Cat# ab32419), anti-N-Cadherin (Abcam Cat# ab76011), anti-E-Cadherin (Abcam Cat# ab40772), anti-Vimentin (Abcam Cat# 92547), anti-p-ERK1/2 (Cell Signaling Technology Cat# 4377), anti-ERK (Cell Signaling Technology Cat# 4697), anti-p-AKT(S473) (Cell Signaling Technology Cat# 9271 ), anti-AKT (Abcam Cat# AB8805), anti-GAPDH (Beyotime Cat# AG019-1), and horseradish peroxidase-conjugated secondary antibodies (Nanjing SunShine Biotechnology Co., LTD.).

### 2.2 Cell Proliferation Assay

A549 or paclitaxel-resistant A549 cells were seeded at 3000 cells/well in 96-well plates and cultured overnight. SphK1 specific inhibitor SKI-II, Cisplatin or paclitaxel were then added to the wells at varying concentrations. The cells were further cultured for additional 3 days. After that, the cell viability was evaluated by using CCK8 kit (Dojindo, Cat# CK04) according to the manufacturer's instructions.

### 2.3 Cell Migration Assay

Cell migration assay was performed using scratch / wound healing test. A549 cells were cultured to 90% confluence in 6-well plates. Scratches were made using a 200μl pipette tip. The cells were then cultured overnight under serum-starved conditions before being supplemented with IGF-1 or SKI-II. Culturing lasted for up to 96 hrs. Images of the cells were taken at selected time points to evaluate the wound-healing condition.

## 3. Results

### 3.1 IGF-1 stimulates SphK1 expression and activation in A549

**Fig 1:**
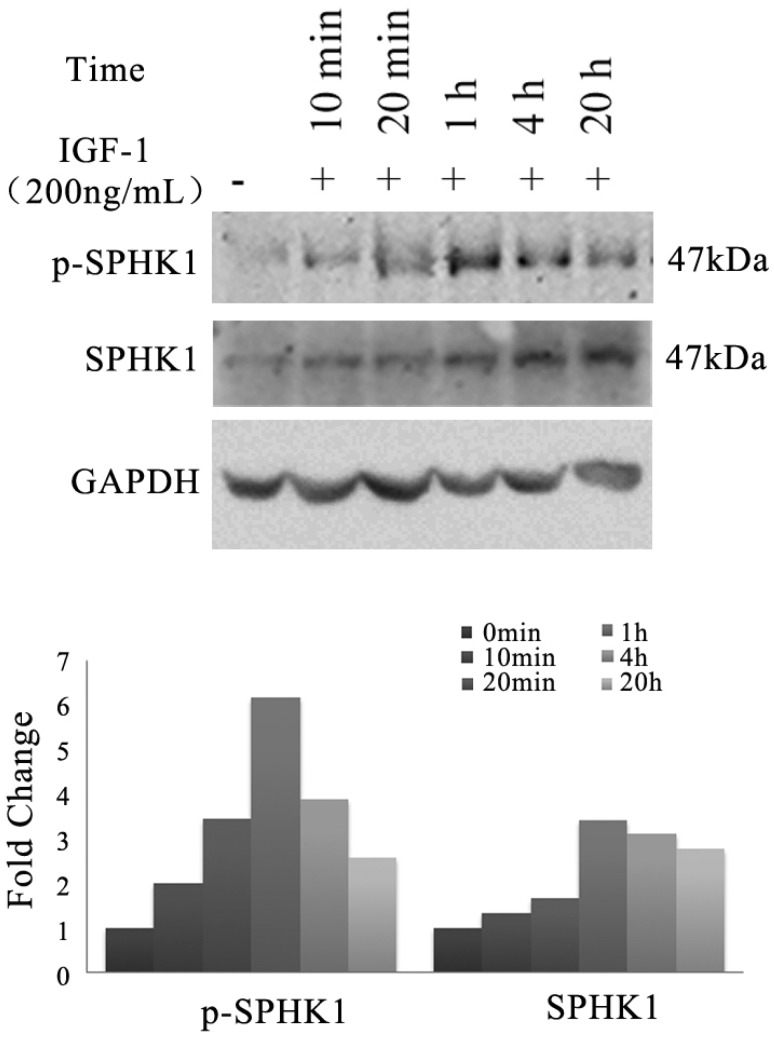
Stimulation of A549 cancer cells with IGF-1 induces overexpression and activation of SphK1. A549 cells were stimulated with 200ng/ml IGF-1 for indicated period of time. Cell lysates were then electrophoresed in 8% PAGE gel and immunoblotted using anti-SphK1 and anti-phospho-SphK1 (Ser225), respectively.

We investigated whether IGF-1 signaling pathway has an impact on the activity of SphK1 in NSCLC cells. A549 cells were treated with 200ng/mL IGF-1, the expression and phosphorylation of SphK1 were examined by Western blot (Figure 1). The results revealed that the expression of SphK1 was increased gradually as early as 20 minutes after IGF-1 stimulation. Meanwhile, phosphorylation of SphK1 at Ser225 was upregulated, which has been shown to increase the catalytic activity and translocation of SphK1 to the plasma membrane, and is crucial for oncogenic signaling 18.

**Fig 2:**
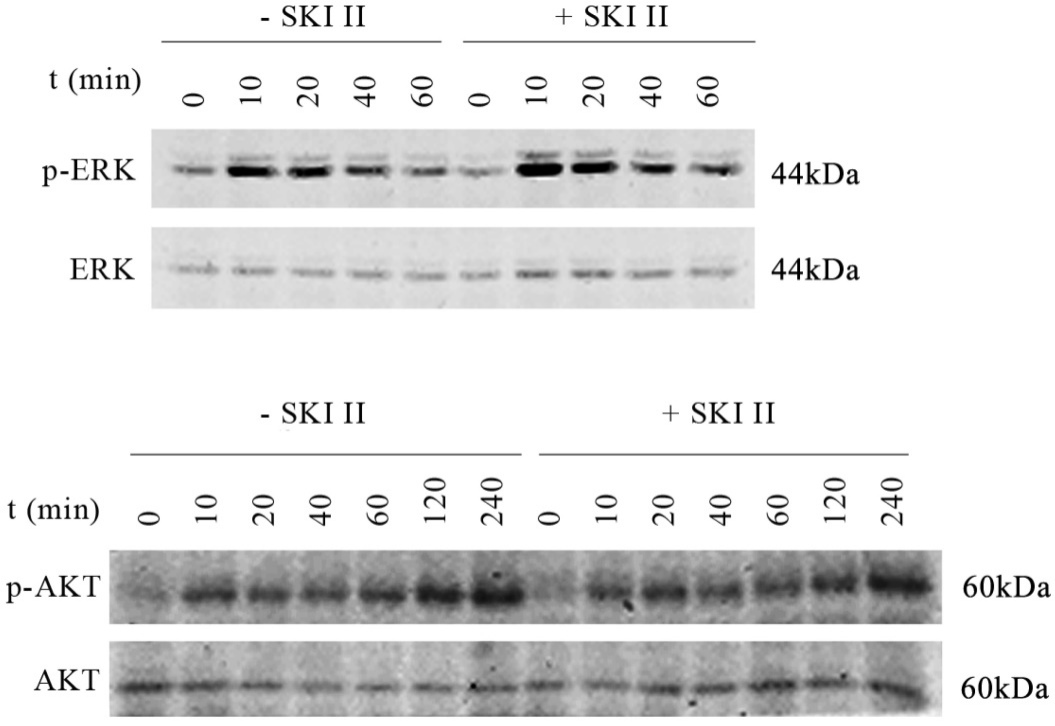
IGF-1 activates both AKT and ERK, treatment of cells with SKI-II has no impact on IGF-1 induced ERK or AKT activation. A549 cells were stimulated with 200ng/ml IGF-1 in the presence or absence of 10μM of SKI-II at various time points. IGF-1 activates both AKT and ERK as indicated by phosphorylation of the proteins. On the other hand, treatment of cells with SKI-II has no impact on the activation of either AKT or ERK.

On the other hand, inhibition of SphK1 activity by its specific inhibitor SKI-II 19 led to minor changes in the activation of either ERK or AKT. Upon IGF-1 treatment, extended activation of phosphorylation of ERK and AKT was indicated (Figure 2). The above results suggest that SphK1 functions downstream of IGF-1 in IGF-1 signaling pathway.

### 3.2 SphK1 activation induces IGF-1-mediated EMT of A549 cells

**Fig 3:**
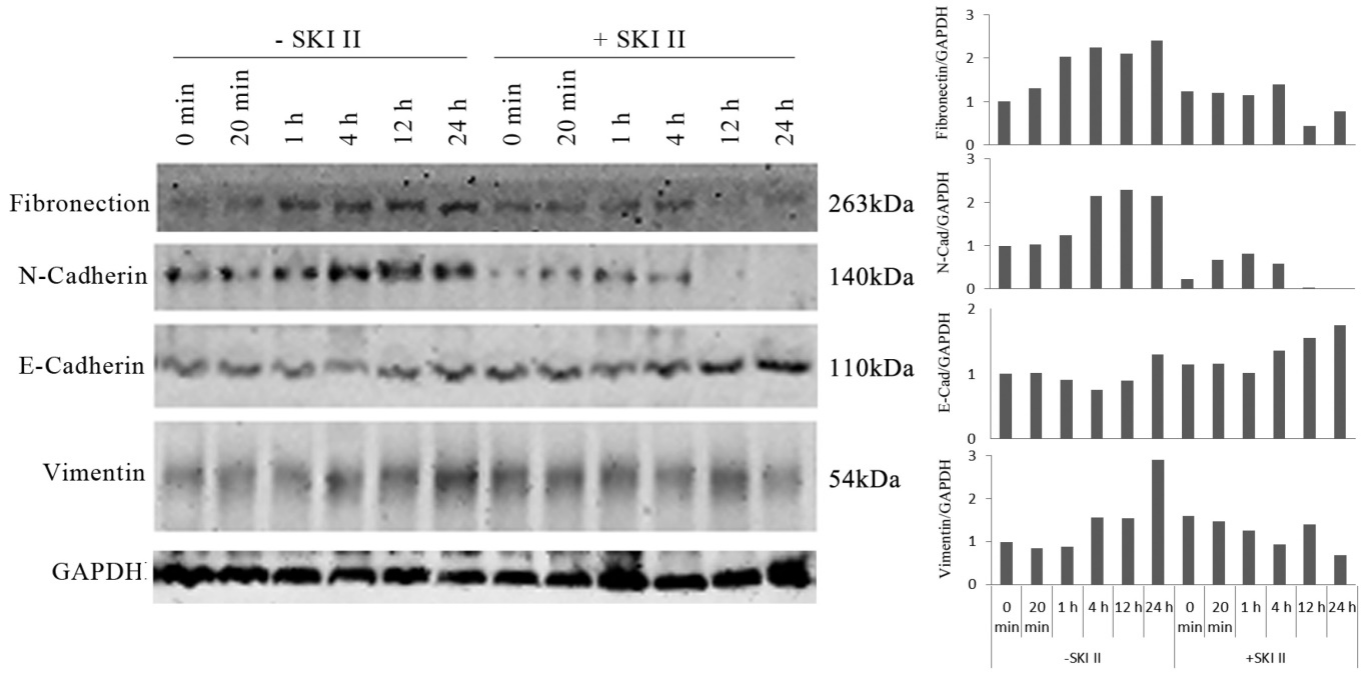
IGF-1-induced EMT in A549 cells was dependent on SphK1 activity. IGF-1 treatment led to decreased E-cadherin expression and increased expression of N-Cadherin, Vimentin and Fibronectin, which are the markers of EMT. Pretreatment of the cells with 10μM of SKI-II reversed the changes of these EMT markers.

We further investigated whether inhibition of SphK1 by its specific inhibitor SKI-II changes the ability of IGF-1 to induce EMT in A549 cells. IGF-1 treatment of A549 cells resulted in changes of EMT biomarkers, including the elevated expression of vimentin, fibronectin, N-Cadherin, and loss of E-cadherin expression. These changes were inhibited by SKI-II (Figure 3). These results suggest that SphK1 activity is critical for IGF-1-induced EMT in A549 cells.

### 3.3 Inhibition of SphK1 alleviates IGF-1-induced cell migration of A549

**Fig 4:**
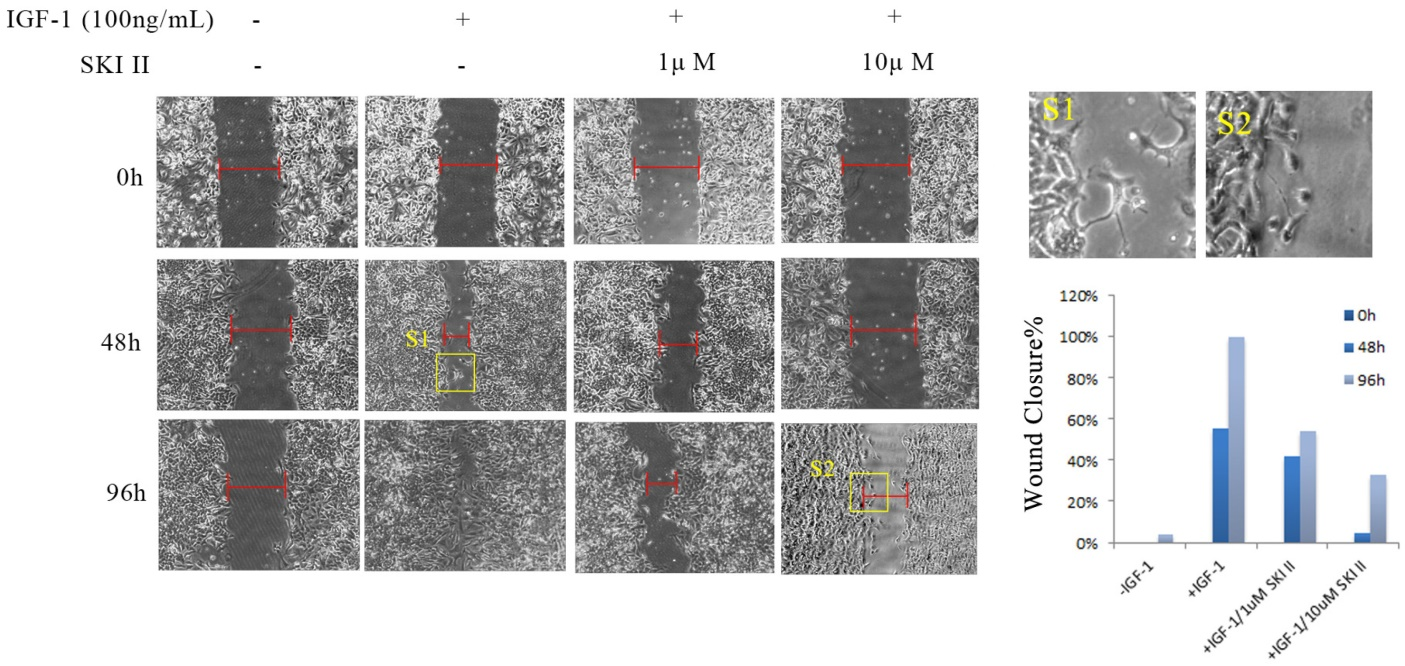
Inhibition of SphK1 suppressed IGF-1-induced cell migration. IGF-1 exhibited potent migratory stimulating activity on A549 cells in a scratch wound healing assay. 96 hours post addition of 100ng/ml IGF-1, the scratch mark almost completed the seal, while little change was seen by 1μM or 10μM SKI-II.

A549 cells were cultured in serum-starved medium, and treatment with IGF-1 at a concentration of 100ng/mL induced apparent cell migration. In a time-course scratch wound healing assay, treating cells with SKI-II at dosages that have no obvious impact on cell proliferation significantly inhibited IGF-1-induced cell migration (Figure 4).

### 3.4 Inhibition of SphK1 increases the sensitivity of A549 cells to paclitaxel

**Fig 5:**
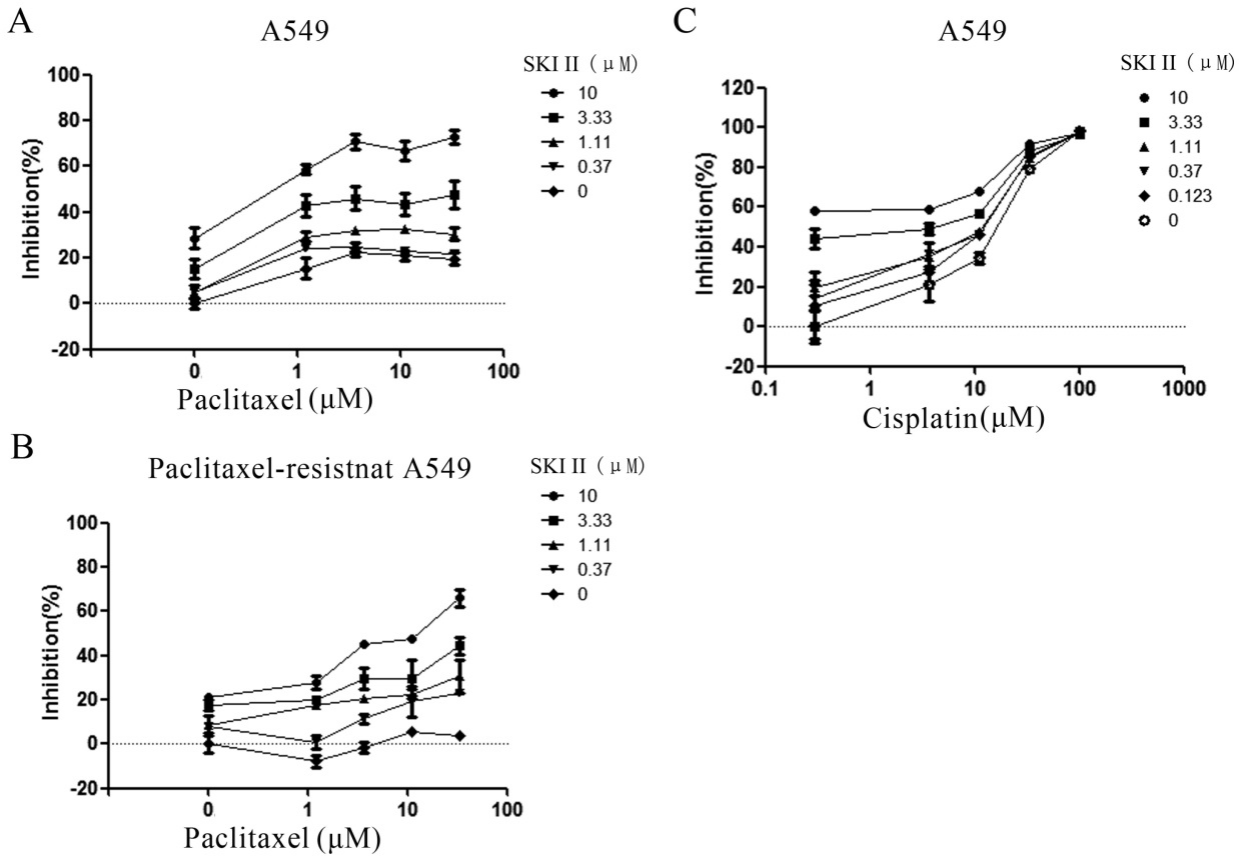
Synergistic inhibition on A549 proliferation by SKI-II and paclitaxel. (A) A549 cells were treated with different combination of SKI-II and paclitaxel as indicated in the figure. Both agents showed dose-dependent inhibitory effects on cell proliferation and synergistic effect was observed in the tested range of concentrations. (B) Paclitaxel alone showed little effect on the proliferation of paclitaxel-resistant A549 cells at as high as 30μM. Addition of SKI-II sensitized the cells to paclitaxel in a dose-dependent manner. (C) No obvious synergistic effect was observed when A549 cell was treated by SKI-II and Cisplatin.

In order to investigate whether SphK1 is involved in the sensitivity of A549 cells to paclitaxel, we evaluated the anti-proliferation effect of paclitaxel in the presence of different concentrations of SKI-II. The results showed that paclitaxel alone only demonstrated very weak cytotoxic effect on A549 cells. Addition of SKI-II enhanced paclitaxel's cytotoxicity in a dose dependent manner (Figure 5A). This synergistic effect was even more evident in paclitaxel-resistant A549 cell line. Treatment of cells with paclitaxel (30μM) showed little cytotoxic effect, while addition of SKI-II significantly restored the sensitivity of the cells to paclitaxel (Figure 5B).

## 4. Discussion

Most of newly diagnosed patients with NSCLC are already at advanced stage, and the prognosis of them remains extremely poor. Therefore, there is a pressing need for optimal therapies 20,21. Mounting evidence has demonstrated involvement of IGF-1-induced EMT in the metastasis and drug resistance, thus contributing to the gloomy prognosis of NSCLC patients 3-6. This scenario promotes the efforts to develop the potential antitumor agents for NSCLC that target IGF-1 pathways.

Our study found that, stimulation of A549 cancer cells with IGF-1 induced overexpression and activation of SphK1, decreased E-cadherin expression and increased expression of N-Cadherin, vimentin and fibronectin, which were dependent on SphK1 activity, while SKI-II, the specific inhibitor of SphK1, could suppress the above effects. These results are consistent with the findings of a previous study, suggesting that SphK1 modulates EMT in colorectal cancer cells 22.

The activation of IGF-I/AKT and IGF-I//ERK pathways contributes to the cell proliferation, migration, invasion, and drug resistance in lung cancer 23-26. Our study showed that IGF-1 activated both AKT and ERK, but, treatment of cells with SKI-II had no impact on IGF-1 induced ERK or AKT activation.

Drug resistance is one of the biggest obstacles to successful treatment and remains a major concern about cancer chemotherapy 27-29. Paclitaxel is a widely used chemotherapy agent for NSCLC treatment. We investigated whether SphK1 activity plays a role in paclitaxel resistance. Cell viability assay showed that enhanced SphK1 activity was involved in paclitaxel resistance of lung cancer cells. Addition of SKI-II enhanced paclitaxel's cytotoxicity in a dose dependent manner, and the synergistic effect was even more evident in paclitaxel-resistant A549 cell line. Interestingly, this mechanism appeared to be drug-related as no synergistic effect was observed when SKI-II worked with Cisplatin (Figure 5C). It is necessary to further investigate what leads to such difference.

A previous study reported that the change of SphK1 expression could affect cell migration, invasiveness and the expression of EMT-related hallmark proteins in A549 cells 15. (Ni, et al., 2015) Our study confirmed the results of this study, and strengthened the evidence base for the role of SphK1 in EMT of A549 cells; Besides, our study uncovered the functional interaction between SphK1 and IGF-1 in the EMT process, and provided new evidence to SphK1 role as a downstream effector of IGF-1.

## 5. Conclusions

In summary, our study demonstrates that, SphK1 acts as a downstream effector of IGF-1 and plays a critical role in IGF-1-induced EMT, migration and paclitaxel resistance of A549 cells, suggesting that SphK1 might be a promising target for the development of a more effective lung cancer therapy, and for the prevention of tumor metastases and drug resistance. Based on this preliminary in vitro study, more in-depth studies are warranted to decipher IGF-1/SphK1 signaling pathway networks and develop potential target specific anticancer agents for NSCLC.

## Abbreviations

AKT: protein kinase B
EMT: epithelial-mesenchymal transition
ERK: extracellular signal-regulated kinase
IGF: insulin-like growth factor (IGF)-1
NSCLC: non-small cell lung cancer
SphKs: sphingosine kinases
SKI-II: SphK inhibitor 2-(p-hydroxyanilino)-4-(p-chlorophenyl) thiazole
